# Molecular Advances in Hypertension and Blood

**DOI:** 10.3390/ijms23010278

**Published:** 2021-12-28

**Authors:** Eleni Gavriilaki, Eugenia Gkaliagkousi

**Affiliations:** 1 Bone Marrow Transplantation Unit, Hematology Department, G. Papanicolaou Hospital, 57010 Thessaloniki, Greece; 2 3rd Department of Internal Medicine, Papageorgiou Hospital, Aristotle University of Thessaloniki, 57010 Thessaloniki, Greece; eugalant@yahoo.com

Hematopoietic cells and their microvesicles have recently emerged as novel markers of cardiovascular risk. The crosstalk between these vesicles and endothelial dysfunction or vascular damage is a field of continuous progress. Additionally, thromboinflammation represents an emerging concept in cardiovascular diseases. In hypertension, the role of signaling pathways in hypertension remains also under investigation. Realizing the unmet needs of increased awareness of treating physicians and active researchers in this complex setting, we launched our Special Issue on “Molecular Advances in Hypertension and Blood”. Our issue has addressed both sides of the coin by publishing four articles that are summarized in this editorial. Firstly, we published an experimental study providing evidence that certain molecular pathways may be involved in myocardial remodeling in the settings of arterial hypertension and chronic kidney disease. Secondly, an in vitro study revealed a novel immune-modulatory effect of Ticagrelor, which is widely used in patients with hypertension and cardiovascular disease. Thirdly, another translational study assessed endothelial injury and pro-coagulant activity using circulating microvesicles in survivors of allogeneic hematopoietic cell transplantation, compared to a control population matched for traditional cardiovascular risk factors. Lastly, a review article delineated the role of Toll-like receptors in the pathogenesis of essential hypertension.

Hematopoietic cells and their microvesicles have recently emerged as novel markers of cardiovascular risk [1,2,3]. The crosstalk between these vesicles and endothelial dysfunction or vascular damage is a field of continuous progress [4]. Additionally, thromboinflammation represents an emerging concept in cardiovascular diseases [5]. In hypertension, the role of signaling pathways in hypertension remains also under investigation. We launched the Special Issue on “Molecular Advances in Hypertension and Blood”, realizing the unmet needs of increased awareness of treating physicians and active researchers in this complex setting. Our Special Issue aimed to address the topic from both sides of the coin, focusing on molecular science and translational studies with biomolecular experiments.

All articles submitted to us for this Special Issue underwent a rigorous peer review process. Ultimately, four articles were published. Three of these articles detail original research into (i) signaling pathways in arterial hypertension and chronic kidney disease, (ii) evidence suggesting a novel immune-modulatory effect of Ticagrelor, and (iii) endothelial injury and pro-coagulant activity in survivors of allogeneic hematopoietic cell transplantation. This Special Issue also includes one review that discusses the role of Toll-like receptors in essential hypertension. These four articles are discussed below.

(i) Bodganova and colleagues aimed to delineate the pathophysiology of myocardial remodeling in arterial hypertension and chronic kidney disease. The authors induced early chronic kidney disease in spontaneously hypertensive rats and compared myocardial remodeling with controls. Chronic kidney disease in these rats was associated with increased FGF23 and decreased renal a-Klotho, along with increased systolic blood pressure, myocardial mass index, cardiomyocyte diameter and myocardial fibrosis. Myocardial remodeling was correlated with increased expression of calcineurin/NFAT and β-catenin. TRPC6 protein levels and mRNA expression were also elevated. Therefore, the authors concluded that Wnt/β-catenin and TRPC6/calcineurin/NFAT signaling pathways may be involved in myocardial remodeling of arterial hypertension and chronic kidney disease, potentially mediated by the FGF23 and α-Klotho axis [6].

(ii) Mitsios and colleagues investigated the effects of Ticagrelor on inorganic polyphosphates released by thrombin-activated platelets and stent-induced neutrophil extracellular traps (NETs) in ST elevation myocardial infarction (STEMI). The authors stimulated neutrophils from healthy individuals and patients receiving Ticagrelor to produce NETs, and further incubated them with plasma from the infarct-related artery of STEMI patients. Then, they assessed the effects of Ticagrelor on NETs and tissue factor loading. Ticagrelor attenuated NETs induced by inorganic polyphosphates and inhibited stent-induced NET release. Considering the clinical need for novel antithrombotic strategies targeting inflammation, their findings highlighted an important and novel immune-modulatory effect of Ticagrelor [7].

(iii) Gavriilaki and colleagues studied 45 survivors of allogeneic hematopoietic cell transplantation (alloHCT) without established cardiovascular disease and 45 control individuals matched for traditional cardiovascular risk factors. Circulating microvesicles (MVs) of different cellular origins (platelet and erythrocyte) were increased in alloHCT compared to controls. Endothelial MVs were increased only in recipients of a myeloablative conditioning. These results suggested that endothelial dysfunction and increased thrombotic risk in alloHCT survivors, independently of traditional cardiovascular risk factors [8].

(iv) Lazaridis and colleagues reviewed the role of Toll-like receptors into essential hypertension considering that subclinical inflammation has been implicated in the pathogenesis of the disease. Toll-like receptors have emerged as novel effectors in the inflammatory background, which is evident from the very early stages of hypertension. This review has highlighted the contribution of innate immunity in the pathogenesis of hypertension and clarified the role of TLR signaling in promoting inflammation and hypertensive vascular damage [9].

Taking into account the multi-disciplinary character of this Special Issue, we hope that it will inspire researchers and clinicians to continue their explorations into novel advances in blood and hypertension.

## References

[1] Gkaliagkousi E., Gavriilaki E., Yiannaki E., Vasileiadis I., Nikolaidou B., Lazaridis A., Dolgyras P., Grigoriadis S., Triantafyllou A., Anyfanti P. (2021). Platelet microvesicles are associated with the severity of coronary artery disease: Comparison between peripheral and coronary circulation. J. Thromb. Thrombolysis.

[2] Gkaliagkousi E., Gavriilaki E., Vasileiadis I., Nikolaidou B., Yiannaki E., Lazaridis A., Triantafyllou A., Anyfanti P., Markala D., Zarifis I. (2019). Endothelial Microvesicles Circulating in Peripheral and Coronary Circulation Are Associated With Central Blood Pressure in Coronary Artery Disease. Am. J. Hypertens..

[3] Gkaliagkousi E., Nikolaidou B., Gavriilaki E., Lazaridis A., Yiannaki E., Anyfanti P., Zografou I., Markala D., Douma S. (2019). Increased erythrocyte- and platelet-derived microvesicles in newly diagnosed type 2 diabetes mellitus. Diabetes Vasc. Dis. Res. Off. J. Int. Soc. Diabetes Vasc. Dis..

[4] Gkaliagkousi E., Gavriilaki E., Triantafyllou A., Douma S. (2015). Clinical Significance of Endothelial Dysfunction in Essential Hypertension. Curr. Hypertens. Rep..

[5] Chrysanthopoulou A., Gkaliagkousi E., Lazaridis A., Arelaki S., Pateinakis P., Ntinopoulou M., Mitsios A., Antoniadou C., Argyriou C., Georgiadis G.S. (2021). Angiotensin II triggers release of neutrophil extracellular traps, linking thromboinflammation with essential hypertension. JCI Insight.

[6] Bogdanova E., Beresneva O., Galkina O., Zubina I., Ivanova G., Parastaeva M., Semenova N., Dobronravov V. (2021). Myocardial Hypertrophy and Fibrosis Are Associated with Cardiomyocyte Beta-Catenin and TRPC6/Calcineurin/NFAT Signaling in Spontaneously Hypertensive Rats with 5/6 Nephrectomy. Int. J. Mol. Sci..

[7] Mitsios A., Chrysanthopoulou A., Arampatzioglou A., Angelidou I., Vidali V., Ritis K., Skendros P., Stakos D. (2020). Ticagrelor Exerts Immune-Modulatory Effect by Attenuating Neutrophil Extracellular Traps. Int. J. Mol. Sci..

[8] Gavriilaki E., Sakellari I., Anyfanti P., Batsis I., Vardi A., Bousiou Z., Lazaridis A., Nikolaidou B., Zarifis I., Masmanidou M. (2020). Assessment of Endothelial Injury and Pro-Coagulant Activity Using Circulating Microvesicles in Survivors of Allogeneic Hematopoietic Cell Transplantation. Int. J. Mol. Sci..

[9] Lazaridis A., Gavriilaki E., Douma S., Gkaliagkousi E. (2021). Toll-like Receptors in the Pathogenesis of Essential Hypertension. A Forthcoming Immune-Driven Theory in Full Effect. Int. J. Mol. Sci..

